# Mobility of LoRaWAN Gateways for Efficient Environmental Monitoring in Pristine Sites

**DOI:** 10.3390/s23031698

**Published:** 2023-02-03

**Authors:** Salma Sobhi, Ahmed Elzanaty, Mohamed Y. Selim, Atef M. Ghuniem, Mohamed F. Abdelkader

**Affiliations:** 1 Wireless Communication Engineering, Information Technology Institute, Ismailia 8366004, Egypt; 2 Electrical Engineering Department, Faculty of Engineering, Suez Canal University, Ismailia 8366004, Egypt; 3 Institute for Communication Systems, University of Surrey, Guildford GU2 7XH, UK; 4 Electrical and Computer Engineering Department, Iowa State University, Ames, IA 50011, USA; 5 Department of Electrical Engineering, Port Said University, Port Said 42526, Egypt

**Keywords:** low-power wide area networks (LPWAN), LoRaWAN, wireless sensor networks (WSN), data collection, mobility, pristine sites

## Abstract

Environmental monitoring of delicate ecosystems or pristine sites is critical to their preservation. The communication infrastructure for such monitoring should have as little impact on the natural ecosystem as possible. Because of their wide range capabilities and independence from heavy infrastructure, low-power wide area network protocols have recently been used in remote monitoring. In this regard, we propose a mobile vehicle-mounted gateway architecture for IoT data collection in communication-network-free areas. The limits of reliable communication are investigated in terms of gateway speed, throughput, and energy consumption. We investigate the performance of various gateway arrival scenarios, focusing on the trade-off between freshness of data, data collection rate, and end-node power consumption. Then we validate our findings using both real-world experiments and simulations. In addition, we present a case study exploiting the proposed architecture to provide coverage for Wadi El-Gemal national park in Egypt. The results show that reliable communication is achieved over all spreading factors (SFs) for gateway speeds up to 150 km/h with negligible performance degradation at SFs=11,12 at speeds more than 100 km/h. The synchronized transmission model ensures the best performance in terms of throughput and power consumption at the expense of the freshness of data. Nonsynchronized transmission allows time-flexible data collection at the expense of increased power consumption. The same throughput as semisynchronized transmission is achieved using four gateways at only five times the energy consumption, while a single gateway requires seventeen times the amount of energy. Furthermore, increasing the number of gateways to ten increases the throughput to the level achieved by the synchronized scenario while consuming eight times the energy.

## 1. Introduction

There has been a growing need for environmental monitoring from governments and businesses in recent years for a variety of applications, including the conservation of sensitive habitats, wildfire containment, and monitoring the quality of life in smart cities. Several works considered implementing effective air quality monitoring systems [1,2,3], water quality [4,5], and pollution levels [6,7,8]. Furthermore, most of the natural resources, such as water, soil, metals, natural gas, and marine and forest habitats, are concentrated in rural areas with limited communication network coverage [9].

Data collection for monitoring environments in rural areas or pristine sites is considered challenging due to the sensitive nature of such sites. Invasive monitoring via fixed communication infrastructure has the potential to alter the natural habitat and alter the attributes or natural behavior of the ecosystem. High communication towers, for example, obstruct migratory birds [10], and infrastructure digging and construction could be hazardous in fossil-rich areas or nature reserves. Moreover, the electromagnetic radiation of such towers near marine reserves compromises the internal compass in marine animals traveling long distances [11,12].

For these reasons, noninvasive methods have been widely proposed for data collection in pristine sites, aiming to leave the environment intact by using infrastructure-free methods. This is commonly achieved via satellite remote sensing or unmanned aerial systems (UAS) [13,14,15]. Environmental monitoring using these methods enables near-real-time monitoring and is constantly integrated with new sensors and technologies. Remote sensing, on the other hand, is almost entirely limited to imagery data sources, with monitoring capabilities limited to thermal or spectral sensing applications such as detecting fires, volcanoes, weather changes, and environmental changes in lands and oceans [16,17].

Noninvasive data collection could also be deployed using low-power wide-area network (LPWAN) protocols due to their long range and simple implementation. Moreover, they have integration capability with all sensor types [18,19]. More importantly, LPWAN protocols such as LoRaWAN are considered green communication protocols because they use less transmission power and have a lower electromagnetic impact on the environment than other communication protocols [20,21,22]. LoRaWAN has been used throughout the literature in many environmental monitoring applications [22,23,24].

In this work, we aim to combine the benefits of the long-range LPWAN protocols with the infrastructure-free nature of UAS remote sensing. We propose using the mobility of LoRaWAN gateways to cover pristine sites by mounting the gateways on top of existing vehicles that are already being used for other purposes in the monitored area. This will increase network coverage without causing unnecessary changes to the ecosystem. The proposed gateway-mounted vehicle collects the monitored data as they pass by the end-nodes along a predefined route and time. The proposed setup can achieve sufficient freshness of information by increasing the frequency of gateway arrivals or the number of mobile gateways collecting data.

Some environmental monitoring applications can endure some information latency [7,25]; however, we consider multiple gateway arrival scenarios in order to achieve a balance between the freshness of information and power consumption. We investigate the performance of three practical scenarios in terms of throughput and energy consumption at different gateway speeds: synchronized, semisynchronized, and nonsynchronized transmission. We also investigate the impact of using multiple gateways on the previously mentioned parameters.

We discuss the effects of LoRaWAN gateway mobility and arrival models on LoRa communication parameters and gateway visibility time. For each gateway arrival scenario, we calculate the number of received packets and the power consumption. We conduct a real-life experiment in which a gateway is mounted on top of a moving car and collects data from an on-ground end-node. The number of packets received for each SF over various gateway speeds is recorded. Finally, we validate our analysis via extensive simulations in both MATLAB and ns-3^®^ network simulations.

We apply our minimally invasive system to monitor one of Egypt’s main natural reserves. Wadi El-Gemal national park is a coastal area along the red sea in south Sinai (24.4747Â° N, 35.0987Â° E). Many researchers are concerned with monitoring the marine and land environment of the area [26,27] as well as the effect of tourism on the natural ecosystem [28]. To preserve the land’s stone nature, in addition to the existing massive mangrove forests and coral reefs, cellular communication towers are only located outside the natural reserve’s boundaries, with many blind spots inside (areas with no cellular coverage). The contributions of this work can be summarized as follows:

1. We propose a novel architecture for a reliable data collection technique for environmental monitoring in pristine sites using a mobile vehicle-mounted LoRaWAN gateway.
2. We analyze different gateway dispatching scenarios and investigate the trade-off between power consumption, end-nodes’ battery life, the freshness of data, and communication reliability.
3. We setup and implement real experiments to validate the derived results and the MATLAB simulation results of all proposed scenarios.
4. We present a case study of the synchronized transmission scenario in Wadi El-Gemal using the ns-3^®^ network simulator.

The rest of the paper is organized as follows. Section 2 gives an overview of some basic features of the LoRaWAN protocol, the effect of mobility on its architecture, and a discussion of related work. Section 3 discusses our proposed system model. Section 4 shows the experiments and results of our simulations. It also presents a simulated case study of Wadi El-Gemal national park using ns-3^®^, one of Egypt’s pristine sites that can benefit from the proposed system. Finally, Section 5 provides the conclusion and future work.

## 2. Background and Related Work

This section provides some background knowledge about the LoRaWAN protocol. Some of the design decisions that will be investigated in our system analysis will be highlighted. We then represent a comprehensive review of the related literature.

### 2.1. Background

**Figure 1:**
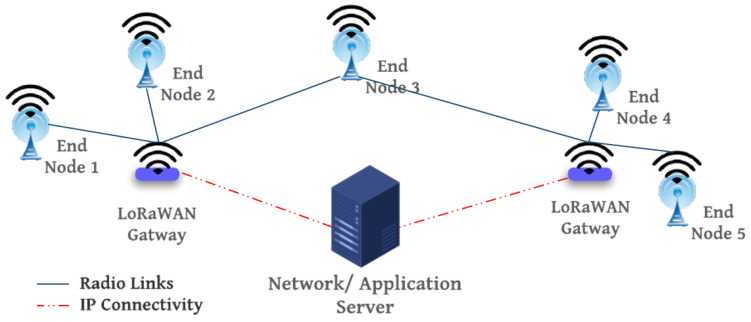
LoRaWAN network architecture.

The LoRaWAN network is formed by a star-of-stars topology as depicted in Figure 1. A group of end-nodes at the bottom level is connected via a single-hop LoRa link to one or many gateways connected over internet protocol (IP) to a common network server and, if needed, to an application server for data storage, processing, and decision-making.

End-nodes collect the required data, attach an end device identifier number, and send it in packets as scheduled regardless of gateway presence; that is, end devices do not need to be associated with a specific gateway to gain network access. In other words, all gateways within range receive the transmitted packets.

LoRaWAN gateways forward them to the network server by encapsulating them in UDP/IP packets that include the received signal strength indicator (RSSI). The network server is then responsible for filtering duplicate and unwanted packets arriving from the same node via multiple gateways and determining which gateway downlink messages are forwarded through. This is performed based on criteria such as range and better radio connectivity [29].

LoRaWAN devices pseudorandomly change their transmission channel in every uplink window. The resulting frequency diversity makes the system more robust against interference. The signals are modulated using a chirp spread spectrum (CSS), with a spreading factor value (SF) that quantifies the number of bits per symbol as follows:

(1) $$N_{c}=\log_{2}\left(\mathrm{SF}\right)$$

This shows that the higher the spreading factor (SF) is, the longer the chirp signal is, hence the further the signal could reach. The choice of (SF) affects the symbol duration and the supported data rate [30]. Furthermore, the bandwidth (BW) is related to the symbol length $l_{s}$ as

(2) $$BW=f_{\max}-f_{min}=\frac{SF}{l_{s}}$$

Each SF leverages the trade-off between transmission rate, reliability, and coverage. Choosing a higher SF value results in a lower data rate and wider coverage. The relation between the input bit rate $R_{b}$, SF, and symbol rate $R_{S}$ is given by

(3) $$R_{b}=R_{S}\;\mathrm{SF}\;$$

The symbol duration in LoRa is

(4) $$t_{s}=\frac{1}{R_{S}}=\frac{2^{\mathrm{SF}}}{\mathrm{BW}}$$

where BW is the bandwidth. The time on air is

(5) $$\begin{array}{cc} \mathrm{TOA} & =t_{s}\;N_{s} \end{array}$$

(6) $$\begin{array}{cc} N_{s} & =N_{p}+N_{\mathrm{pl}} \end{array}$$

where $N_{s}$ is the number of symbols, $N_{p}$ is the of preamble symbols, and $N_{pl}$ is the number of payload symbols [31].

**Figure 2:**
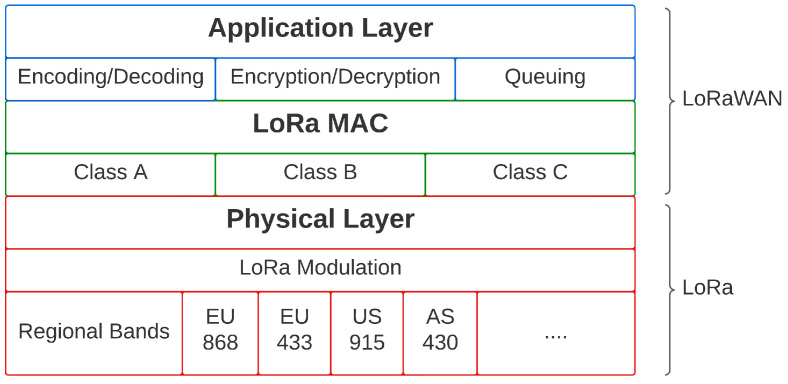
LoRaWAN protocol stack.

LoRaWAN MAC layer defines three classes of end devices for different operation needs. Classes A, B, and C are shown in Figure 2, where the key difference is their trade-off between communication latency and power consumption [32,33]. With gateway mobility, the selection of LoRaWAN operation class and SF should be considered to compromise between power consumption limitations and increasing the probability of gateway detection. In this work, we use LoRaWAN to utilize its off-the-shelf devices and software without needing any firmware modification.

ALOHA is used for medium access, and each end-node starts its transmission window with a random time offset. Collisions occur when two nodes are transmitting simultaneously on the same frequency using the same SF. If a collision occurs and transmission fails, the transmitter waits for an indeterminate amount of time before retransmitting. Due to the limited gateway availability and the standard requirements of *D* airtime duty cycle for end-nodes, this could result in a significant time delay, which would be intolerable in the case of mobility.

### 2.2. Related Work

LoRaWAN has been incorporated repeatedly in environmental monitoring applications. For example, in [34], the authors present a real-time system for monitoring temperature, humidity, and solar radiation in agricultural lands in rural Indonesia where cellular coverage is unavailable; their system has successfully transferred data from sensing nodes to gateways on a maximum distance of 800 m with less than 20% packet loss. Analysis of using low-power area networks such as LoRaWAN in agriculture was presented in [35]. To monitor and control farm assets and infrastructure, the authors employed a cloud-based LoRaWAN platform, proving that LoRaWAN is superior to other long-range communication standards for resource management and monitoring in large-scale farms.

Utilizing LoRaWAN connectivity in sensitive habitats was demonstrated in [23]. The authors monitored oyster aquaculture in Australia’s Clyde River, where atmospheric and marine temperature and salinity sensors are attached to river buoys. The sensors collect data to help farmers make management decisions and prevent the loss of oyster leases due to extreme weather events. LoRa-based ecological water quality monitoring systems in aquaculture were presented in [36,37]. Using LoRa technology allows for low-cost, simple to develop and deploy systems that are efficient and environmentally friendly.

Applying mobility to the LoRaWAN networks enhances the efficiency of monitoring systems in many domains. The authors of [38] applied various mobility models under LoRaWAN channel and investigated communication quality. According to their findings, the effect of mobility on the LoRaWAN link is nearly limited to the Doppler effect and the increased number of collisions as the number of mobile end-nodes increases.

In [39], end-nodes mobility was leveraged to extend the sensing area by implementing sensors for toxic and inflammable gasses in wearable devices carried around by workers in a factory in Italy. The system achieved near-real-time performance by maintaining latency less than 500 ms and almost 100% packet delivery rate (PDR) for more than 200 end-nodes; the end-nodes were clustered by location and differentiated into subbands to minimize collision and transmit efficiently while complying with the standard duty cycle.

Mobility of LoRaWAN gateways was exploited in [40]. Drones equipped with LoRaWAN gateways were used to detect fires in forests, where a network of ground infrared cameras was set up to monitor any thermal changes in the area. LoRaWAN gateways would fly over the target areas collecting camera readings before returning to offload the data into a server for early fire detection. The same approach was implemented in [41] to guarantee rapid detection of environmental parameters in infrastructure-less areas. Most of the mentioned work used specialized drones, which are costly and have limited power. Instead, we propose utilizing the existing traveling vehicles for data collection using LoRaWAN gateways.

To ensure seamless connectivity in mobile scenarios, issues such as Doppler effects during movement [42], uplink and downlink communications, and adaptive data rate (ADR) technique must be studied. Moreover, the handover of an end-node from the coverage of a gateway to another and end-node localization should be taken into consideration [43,44]. An advanced SF selection scheme for mobile end-nodes was presented in [45]; SFs were assigned to the end-nodes by proactively responding to their mobility in a method that increased the PDR in comparison with the LoRaWAN-associated ADR.

Mobility in LoRaWAN networks where the gateway is fixed and end-nodes are mobile is extensively studied. This is expected due to the wide range of IoT applications in smart wearable devices, vehicle communication, and tracking devices that benefit from this scenario. The authors in [46] reviewed various IoT systems for mobility support and concluded that mobility in LoRaWAN can be easily achieved in uplink while operating in any of the three classes, with the expense of some latency in downlink due to the time needed by the network server to update its forwarding table as the end-nodes change their location.

Some field tests for end-nodes mobility in urban, suburban, and rural areas were conducted in [42]. We are most concerned with the measured PDR in the rural scenario where the end-node traveled at a speed of 32.5 km/h along a route 20 km away from the gateway and achieved 96% PDR at a distance of 18.5 km using SF12 and 100% PDR at 9.6 km using SF7. The authors of [47] conducted extensive measurements on end-node mobility at relatively high speeds. Their analysis of received packet percentage and received signal strength demonstrates the robustness of LoRaWAN communication with mobility, with little degradation in speeds exceeding 90 km/h when compared to fixed setups.

In the opposite scenario, where the end-nodes are fixed and the gateway is mobile, the work carried out is almost limited to unmanned aerial systems that use LoRaWAN gateways as mobile data sinks for various applications such as localization [48,49], the backup communication system in case of environmental disasters or hostile locations such as mines and forests [50,51], and many environmental monitoring applications. The performance analysis of such systems is mainly concerned with the efficient scheduling of the data collection and routing of the LoRaWAN gateway due to the limited power capacity in UASs [52,53,54]. Unmanned aerial systems collect data while hovering over the end-nodes at low speeds and stay in location until all transmission is complete. In comparison, our work focuses on analyzing LoRaWAN communication over relatively high speeds and limited gateway availability.

The mobility of LoRaWAN gateways requires an appropriate choice of spreading factor and LoRa configuration. One option is to use higher spreading factors to ensure stable communication through the vertical distance between the ground end-nodes and the drone hovering over them in [41,55]. However, using lower spreading factors with a shorter time on air is more suitable for tracking applications and real-time communication when shorter intertransmission time is required [49,51].

Experiments of the coverage and speed limits of mobile LoRaWAN gateways in the literature showed successful LoRaWAN communication on speeds of 10–50 km/h and vertical distances of 30–300 m [5,56,57,58], which is to be expected due to the limitations in power in the unmanned aerial systems used in these experiments. Our previous work [59] presents real experiment results and performance analysis of the mobility of LoRaWAN gateways with relatively much higher speeds, showing that transmission can be successful with SFs 7 to 10 at speeds up to 160 km/h.

## 3. Methodology

This section explains the proposed LoRaWAN mobility-assisted data collection model. The effect of gateway mobility on LoRaWAN communication is investigated. We examine system parameters such as range, maximum throughput, and power consumption. We present various gateway arrival scenarios to improve data freshness, and we analyze the optimal wake-up schedule for the end-node in order to guarantee the presence of the gateway in each scenario while conserving end-node power.

### 3.1. System, Signal, and Channel Models

**Figure 3:**
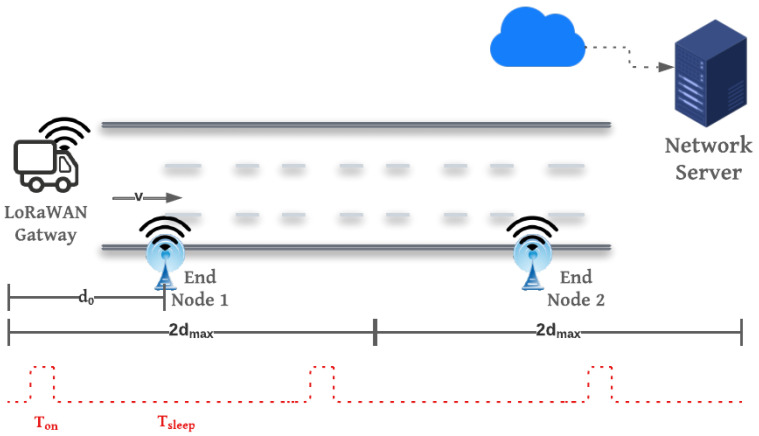
Proposed system model.

We consider rural and pristine areas with minimal IoT infrastructure, as well as delay-tolerant monitoring applications that require infrequent data transmission. The proposed system, depicted in Figure 3, is composed of a vehicle equipped with an LoRaWAN gateway integrated with a GPS module, end-nodes distributed along the highway, and a cloud-based LoRaWAN network and application servers with access to other communication network coverage.

The end-nodes are remotely located and transmit packets sporadically throughout the day on one or more preconfigured time slots. To save power, the end-nodes open transmission windows at a predetermined rate per hour ($R_{on}$) to sense the presence of the gateway before data transmission. Once the gateway is detected, data transmission time should not exceed the gateway visibility time ($T_{v}$), which is defined as the total time at which the end-nodes and gateway are in range. In that sparse deployment of end-nodes in remote areas, we consider that the nodes are distanced enough that only one node is transmitting within the gateway visibility time ($T_{v}$).

The gateway travels with speed *v*, collects end-nodes data, and offloads these data to the network/application server when the gateway reaches a location with an internet connection. The gateway is integrated with a GPS module for localization purposes in downlink communication. Three gateway arrival scenarios are investigated in this model. Each scenario provides a varying degree of flexibility in the data collection schedule at the cost of a trade-off between power consumption and communication reliability. These scenarios are synchronized transmission, semisynchronized transmission, and nonsynchronized transmission.

We investigate the performance of our proposed system from two main aspects: the effect of gateway mobility on the LoRaWAN signal in terms of the Doppler effect, maximum coverage range, the limited availability of the gateway, and maximum throughput; and the effect of different gateway arrival scenarios on the availability of the gateway, maximum throughput, freshness of data, and power consumption.

#### 3.1.1. Mobility Consideration: Doppler Shift

LoRa modulation is a proprietary spread spectrum method based on chirp spread spectrum modulation, which improves the sensitivity of the receiver by giving tolerance for frequency mismatch between the transmitter and receiver. In addition, it makes LoRa somehow resistant to fading, multipath interference, and Doppler effects. However, for mobile gateways, the Doppler shift, $f_{D}$, should be taken into consideration.

To inspect the Doppler shift appearing as a small frequency shift on the LoRa signal at the receiver, the channel coherence time $t_{c}$, which is the time duration within which the channel remains constant, and symbol duration $t_{s}$ should be compared [42]. Coherence time is inversely proportional to Doppler spread, i.e.,

(7) $$t_{c}=\frac{1}{f_{D}}.$$

When the symbol time of LoRa is larger than the coherence time, the amplitude and phase changes imposed by the channel vary considerably during the transmission of one symbol. To cope with the Doppler spread, the system should be designed such that $t_{s}<t_{c}$.

**Figure 4:**
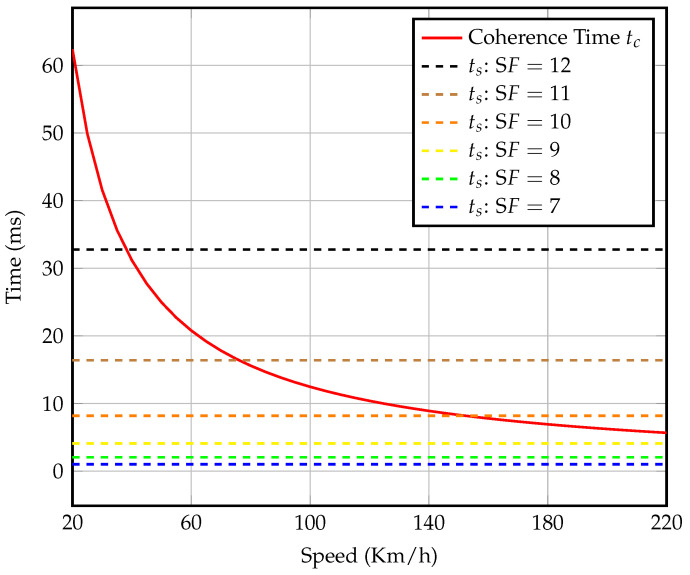
Symbol times for different spreading factors and coherence time with mobility.

In this regard, we inspect the coherence time and the symbol duration for various gateway speeds and spreading factors, respectively, as shown in Figure 4. This figure considers a center frequency of 868 MHz and 125 kHz bandwidth. We conclude that transmission with SFs up to 10 is less sensitive to Doppler shift if the gateway speed is below 160 km/h; however, for higher SFs, this speed limit is reduced to 80 km/h. On the other hand, higher SFs implies a longer communication range that increases the visibility time for moving gateway at higher speeds. Therefore, there is a trade-off between the selection of lower SFs to ensure reliable communication and the selection of higher SFs to enhance the communication range.

#### 3.1.2. Mobility Consideration: Spreading Factor Allocation and Visibility Time

We define the visibility time at a certain speed as twice the maximum transmission time, assuming that the transmission starts as the gateway approaches the end-node from a distance, passes by the node, and then moves away in the opposite direction, i.e.,

(8) $$T_{v}=\frac{2\;d_{\max}}{v}$$

In the case of a moving gateway, selecting SF value must be optimized to ensure time on air (TOA) that is long enough to transmit all data yet is still below both the $t_{c}$ and $T_{v}$ boundaries.

To study the effect of these boundaries on the maximum throughput for each SF, we need to *(i)* calculate the maximum distance that the signal could travel under the path loss conditions, and *(ii)* calculate the maximum visibility time depending on the gateway moving speed and the time at which transmission starts at the end-node, while taking into account the transmission duty cycle. The bounds of transmission distance and visibility time for each SF and different gateway speeds are listed in Table 1. In the remainder of the paper, we consider the maximum speed of the gateway such that the Doppler effect is minimal.

#### 3.1.3. Path Loss Model

For the path loss model, we consider the Okumura–Hata propagation model [60]. This propagation model is commonly used for rural scenarios due to its compatibility with macrocell characteristics. The path loss for the Okumura–Hata is written as

(9) $$P_{l}\left(d\right)=A\left(f_{c},h_{b},h_{m}\right)+B\left(h_{b}\right)\;\log\left(d\right)-C\left(f_{c}\right)$$

where $f_{c}$ is the carrier frequency, $h_{b}$ and $h_{m}$ are the antenna heights in kilometers at the base station (gateway) and end-node, respectively, and *d* is the link distance in kilometers. The functions $A(f_{c},h_{b},h_{m})$, $B(h_{b})$, and $C(f_{c})$ depend on the transmission setup of carrier frequency and antenna heights as well as other constants relevant to the environment, and these constants can represent the channel model for a rural scenario, as detailed in [60].

The maximum communication distance, $d_{\max}$, is computed as the maximum possible distance such that the received power $P_{r}$ is higher than the receiver sensitivity $P_{rs}$, as shown in the first row of Table 1. We calculate $d_{\max}$ using the Okumura–Hata discussed above as:

(10) $$\begin{array}{ccccccc} \; & d_{\max}=\underset{d\in R}{\mathrm{argmax}} & \; & d\; & s.t. & \; & P_{r}\left(d\right)≜P_{t}-\;P_{l}\left(d\right)\geq P_{rs}\left(\mathrm{SF}\right) \end{array}$$

where $P_{t}$ is the transmit power.

### 3.2. Gateway Arrival Scenarios

In this section, we present different gateway arrival scenarios in order to compromise between reducing power consumption and ensuring the freshness of collected data. This is achieved by investigating different levels of synchronization between the end-node transmission and the gateway arrival, i.e., synchronized, semisynchronized, and nonsynchronized end-node transmission. Synchronized transmission can be achieved practically by using a scheduled vehicle, such as a bus, that travels a specific route on a strict time schedule. Semisynchronized transmission attempts to relax the strict time constraint by allowing for some delay time in the gateway’s arrival, while the nonsynchronized arrival model is better suited for collecting data from unpredictable traffic. For each scenario, we study the effect of synchronization on power consumption and throughput.

#### 3.2.1. Synchronized Transmission

**Figure 5:**
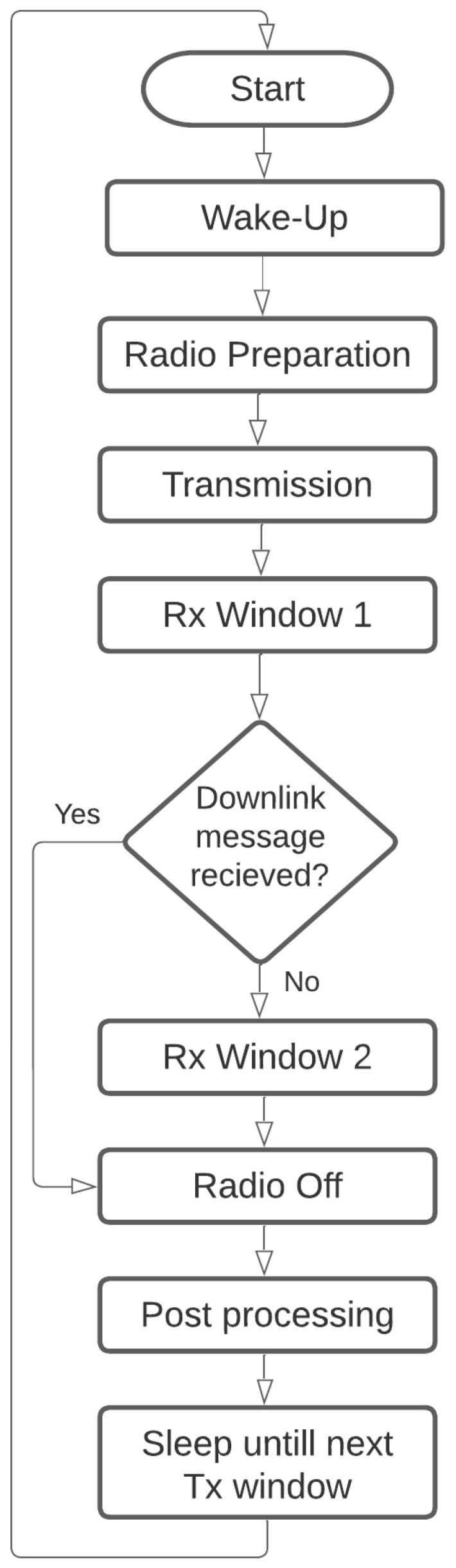
End-node procedure in synchronized transmission.

This scenario assumes perfect synchronization between the start of end-node transmission and the time the gateway arrives into range. This setup necessitates precise gateway arrival scheduling or some sort of synchronization method to wake up the end-nodes at the time of gateway arrival. This maximizes the throughput and minimizes the power consumption as the end-nodes are awake only during transmission time. On the other hand, because data could only be transmitted during prescheduled transmission windows, this provides the least freshness of data performance. The flow diagram in Figure 5 shows the transmission procedure at the end-nodes.

In the case of perfect synchronization, the end-node is assumed to begin transmission once the gateway enters the end-node communication range, that is, when the gateway is at a distance $d_{0}$ = $d_{\max}$ away from the end-node, as depicted in the system model shown in Figure 3. The maximum number of transmitted packets of a certain payload $K_{pl\;}$ is the total number of packets to be transmitted within the visibility time $T_{v}$ of a certain gateway speed *v* while applying the 1% duty cycle (D) constraint regulated by LoRaWAN standard, i.e., for each transmission, the end-node must wait for a time window equal to $\frac{(1-D)\;\mathrm{TOA}}{D}$ before the next transmission [29].

The maximum number of transmitted packets in the case of synchronized transmission $\left(K_{pl}\mathrm{ST}\right)$ is calculated such that the total transmission time of TOA and the corresponding waiting time is within the visibility time, as

(11) $$K_{pl}^{\mathrm{ST}}\;\left[\mathrm{TOA}+\frac{(1-D)\;\mathrm{TOA}}{D}\right]\leq T_{v}$$

There is no need to account for the waiting time for the last transmitted packet, hence

(12) $$\left(K_{pl}^{\mathrm{ST}}-1\right)\;\frac{\mathrm{TOA}}{D}+\mathrm{TOA}\leq T_{v}$$

With a simple rearrangement of variables and applying the floor function, the expression becomes

(13) $$K_{pl}^{\mathrm{ST}}=\left\lfloor \frac{D(T_{v}-\mathrm{TOA})}{\mathrm{TOA}}\right\rfloor +1$$

This expression takes into account the total transmission time for each packet TOA as well as the uplink transmission duty cycle, D=0.01. The value of TOA for a certain payload length and a certain LoRaWAN packet configuration is calculated as in [61].

#### 3.2.2. Semisynchronized Transmission

**Figure 6:**
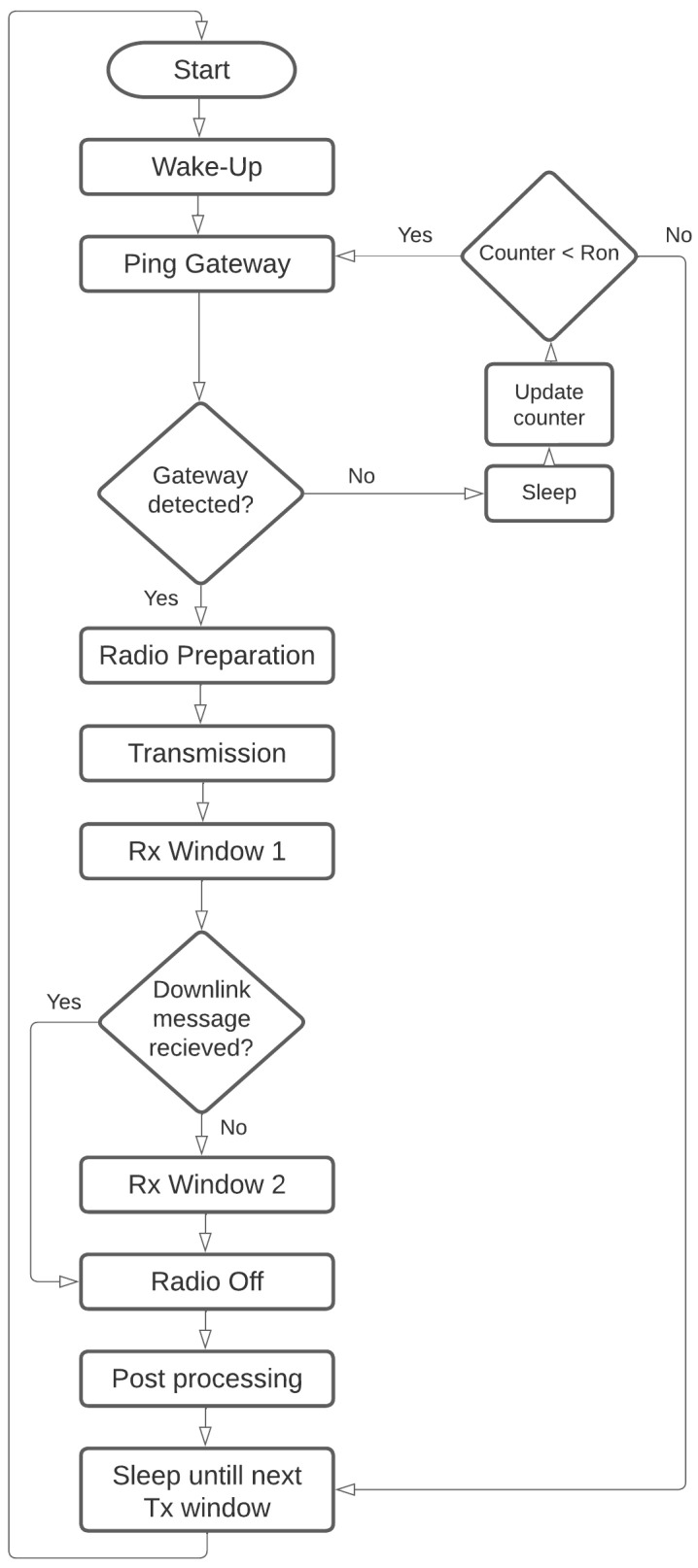
End-node procedure in semisynchronized and nonsynchronized transmission.

In this scenario, we aim to relax the synchronization constraint. At the end-node side, the gateway is expected in a longer time window, such as one hour. As a result, there is some transmission delay tolerance. This configuration consumes more power because the end-nodes must now wake up for a longer period of time, as illustrated in Figure 6. The end-node anticipates the gateway arriving during a certain time window, but it does not know the exact gateway arrival time.

In order to save power, the end-node can open the receive window periodically, i.e., $R_{\mathrm{on}}$ times per hour, to search for the gateway beacon. This will cause a reduction in the visibility time by a random amount, i.e., $t_{x}$. The reason is that the actual transmission starts when the gateway is at distance $d_{0}=d_{\max}-vt_{x}$ instead of $d_{\max}$.

To analyze the effect of this random delay on the total throughput, we define $t_{x}$ as a uniformly distributed random variable over $\left[0,T_{\max}\right]$, where $T_{\max}$ is the time difference between two consecutive receive windows, representing a reduction in the visibility time, i.e., $T_{\max}=3600/R_{\mathrm{on}}$ in seconds. We should consider that $T_{\max}$ < $T_{v}$, to ensure that we do not miss the presence of the gateway.

The instantaneous maximum number of transmitted packets for semisynchronized transmission ${K_{pl}}^{\mathrm{SS}}$ should account for the random reduction in visibility time, as

(14) $${K_{pl}}^{\mathrm{SS}}=\left\lfloor \frac{D(T_{v}-t_{x}-\mathrm{TOA})}{\mathrm{TOA}}\right\rfloor +1.$$

The average number of transmitted packets can be lower-bounded as

(15) $$E\left[{K_{pl}}^{\mathrm{SS}}\right]\geq E\left[\frac{D(T_{v}-t_{x}-\mathrm{TOA})}{\mathrm{TOA}}\right]=\frac{D(T_{v}-0.5T_{\max}-\mathrm{TOA})}{\mathrm{TOA}}$$

where $E\left[t_{x}\right]=0.5T_{\max}$.

#### 3.2.3. Nonsynchronized Transmission

In this final setup, we aim to completely eliminate the synchronization constraint. As in real-life scenarios, it is difficult to specify the exact arrival time of the gateways. End-nodes could transmit once the data are ready and the gateway is detected without committing to a transmission schedule. Multiple gateways can be used to improve the probability of detection of a gateway at any time of day. The drawback is that excessive power is consumed at the end-node side while attempting to detect a gateway and begin transmission. In this section, we investigate the optimum end-node wake-up schedule to improve the probability of gateway detection and throughput while minimizing power consumption.

Multiple gateways are passed by the end-nodes at random arrival times in this scenario. Instead of assuming a fixed time of arrival with a random delay, the gateway’s arrival time $t_{a}$ is completely random. We assume that $t_{a}$ is uniformly distributed over a number of hours *H*. The probability for a gateway arrival in any second is similar to the previous case, but with $T_{\max}=H\times 3600$ s. To detect the presence of a gateway, the end-node continues to open receive windows with fixed size $T_{\mathrm{on}}$ as shown in Figure 3 at a rate per hour $R_{\mathrm{on}}$ along the arrival time frame. The actual transmission starts when the arrival time resides in one of these slots with a probability

(16) $$P_{T_{X}}=\left\{\begin{array}{cc} \frac{T_{\mathrm{on}}\;R_{\mathrm{on}}\;H}{T_{\max}} & \mathrm{for}\;t_{a}\in \left[0,T_{\max}\right]\\ 0 & \mathrm{otherwise} \end{array}\right.$$

with $T_{\mathrm{on}}\;R_{\mathrm{on}}\;H\leq T_{\max}$, to minimize the opening rate for power-saving. Using multiple gateways $N_{G}$ increases the probability of gateway detection to

(17) $$P_{T_{X}}=\left\{\begin{array}{cc} \frac{N_{G}\;T_{\mathrm{on}}\;R_{\mathrm{on}}\;H}{T_{\max}} & \mathrm{for}\;t_{a}\in \left[0,T_{\max}\right]\\ 0 & \mathrm{otherwise} \end{array}\right.$$

Throughput is affected by the probability of detection, the number of gateways, and the number of transmission windows. The maximum throughput for the nonsynchronized case ${K_{pl}}^{\mathrm{NS}}$ with multiple gateways can be expressed as

(18) $${K_{pl}}^{\mathrm{NS}}={K_{pl}}^{\mathrm{ST}}\;P_{T_{X}};$$

however, when the gateway presence is detected, its position at the start of transmission is at $d_{0}$, as explained in the previous scenario; hence, we could express the average throughput for nonsynchronized transmission as

(19) $$E\left[{K_{pl}}^{\mathrm{NS}}\right]=E\left[{K_{pl}}^{\mathrm{SS}}\right]\;P_{T_{X}}$$

It is worth mentioning that this scenario can offer better performance regarding the freshness of the data. Increasing the number of gateways decreases the latency as the end-node is always searching for gateways when the data are ready [62]. In contrast to the other scenarios, the freshness of data is at its best only for the data generated in the limited time of the gateway arrival.

### 3.3. Power Consumption Model

In our proposed system, the gateway is powered by the vehicle on which it is mounted via USB, and the end-nodes are battery-powered. This section aims to analyze the power consumption of end-nodes in order to maximize battery life. The end-nodes are only awake during scheduled transmission windows. This requires precise scheduling of end-nodes transmission and the presence of the moving gateway to achieve maximum power-saving and preserve the ecological nature of the site. The spreading factor used for transmission greatly affects power consumption, as the TOA nearly doubles for every higher SF. Thus, the power consumption and battery drainage increase. The daily energy consumption can be calculated as

(20) $$E=E_{\mathrm{On}-\mathrm{Off}}+E_{\mathrm{Search}}+E_{\mathrm{Transmit}}+E_{\mathrm{Receive}}$$

such that $E_{\mathrm{On}}-\mathrm{Off}$ is the power consumption for wake-up and sleep states, which is repeated depending on the frequency of data collection as

(21) $$E_{\mathrm{On}}-\mathrm{Off}=V\;\sum_{n=1}^{N}\left(T_{\mathrm{WU}}\;I_{\mathrm{WU}}+T_{\mathrm{RP}}\;I_{\mathrm{RP}}+T_{\mathrm{TS}}\;I_{\mathrm{TS}}+T_{S}\;I_{S}\right)$$

where *V* is the input voltage, usually equal to 3.3 V, *N* is the number of data collection times per day, and $I_{x}$ is the drained current for a certain state for time duration $T_{x}$. Table 2 lists the detailed parameters of the energy consumption model used in this work, with more details to be found in [63].

$E_{\mathrm{Search}}$ is the energy consumed by the end-node while searching for the gateway presence; this state only occurs in the semisynchronized and nonsynchronized scenarios, and its maximum value is calculated as

(22) $$E_{\mathrm{Search}}=V\;\sum_{n=1}^{R_{on}\;H}T_{\mathrm{PS}}\;I_{\mathrm{PS}}$$

where $E_{\mathrm{Search}}=0$ for the synchronized transmission scenario and H=1 for the semisynchronized scenario.

$E_{\mathrm{Transmit}}$ is the energy consumed for data transmission, which is dependent on the used SF and the number of transmitted packets $K_{pl}$, while $E_{\mathrm{Receive}}$ is the energy consumed while opening the two receive windows and processing downlink messages.

(23) $$E_{\mathrm{Transmit}}=V\;\sum_{n=1}^{N}\left(T_{X}\left(K_{pl},\mathrm{SF}\right)\;I_{X}+T_{\mathrm{RO}}\;I_{\mathrm{RO}}+T_{\mathrm{PP}}\;I_{\mathrm{PP}}\right)$$

The summation limit for each state depends on the transmission model and the number of trails, as shown in Figure 5. The transmission procedure is straightforward, each state occurs once, and the main factor in the energy consumption is the transmission state, which depends on the used SF, and the number of packets transmitted. However, in Figure 6, the process of gateway search consumes a significant amount of energy, which increases with high $R_{on}$ values or low probability of detection. Moreover, there is a worst-case scenario where the gateway is not detected. This dramatically increases the energy consumption values for semisynchronized $E^{\mathrm{SS}}$ and nonsynchronized transmissions $E^{\mathrm{NS}}$.

## 4. Results and Discussion

In this section, we validate our analysis by the means of three different methods: *(i)* a theoretical performance analysis is conducted; *(ii)* extensive simulation for the proposed system with different gateway arrival scenarios, i.e., synchronization conditions; *(iii)* real-world experiment is carried out to assess the effect of gateway mobility on throughput. Furthermore, to test the practicability of our proposed scheme, we propose a case study in which we test the proposed system in the Wadi El-Gemal area by running ns-3^®^ simulations.

Real Experiment Setup

**Figure 7:**
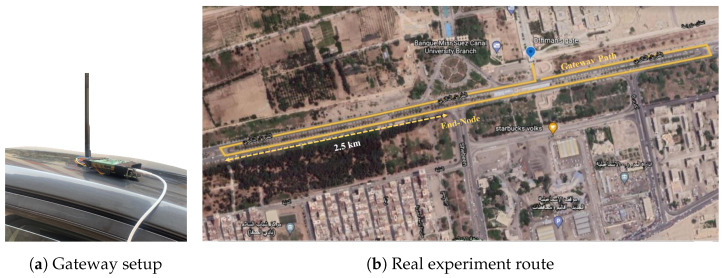
The experiment setup.

For the real experiment, the gateway was built using an SX1301 Semtech^™^ transceiver [64]. The SX1301 transceiver is controlled by a Raspberry Pi II board, which also acts as an LoRaWAN network server and temporary data storage. The Raspberry Pi is later connected via IP protocol to the application server created on Chirpstack^®^ [65]. For the end-node, module SX1278 [66] is used, with 868 MHz antenna of gain 2.5 dBi. The end-node is placed on the ground alongside an empty road with a clear line of sight to the gateway at an initial distance of 2.5km. The end-node transmits 25 packets using each SF to the gateway. The gateway is moving inward with speed $v\in \left[60:120\right]\;\mathrm{km}/h$ and continues straight until the node is out of range. Figure 7 shows the real experimental setup, where the gateway is placed on top of a car traveling down the road parallel to the Suez Canal University campus in Ismailia, Egypt.

Simulation Setup

The same setup used in the real experiment was applied in simulations. The simulation setup parameters for the MATLAB and ns-3^®^ network simulations, such as antenna heights, gateway speed, initial distance, and packet configurations, are listed in Table 3. The Okumura–Hata model was used as a propagation model in all simulations. For ns-3 simulations, C++ scripts were written to configure the LoRaWAN module and mobility module. The total number of symbols in an LoRaWAN frame after coding and the corresponding TOA are calculated as in [31].

### 4.1. Effect of Mobility on the Performance Measures

In this experiment, we validate our analysis of the effect of gateway mobility on the LoRaWAN communication link and the total number of correctly received packets. We compare the number of received packets recorded in the actual experiment setup to the numbers indicated by both analytical results calculated via (13) and simulation results via ns-3 simulation. The number of correctly received packets is calculated as the number of successfully decoded packets at the gateway in both the real and simulated experiments. However, in the analytical results, the number of received packets considered is the number of received packets whose received signal strength (RSSI) is greater than the receiver sensitivity for each SF.

**Figure 8:**
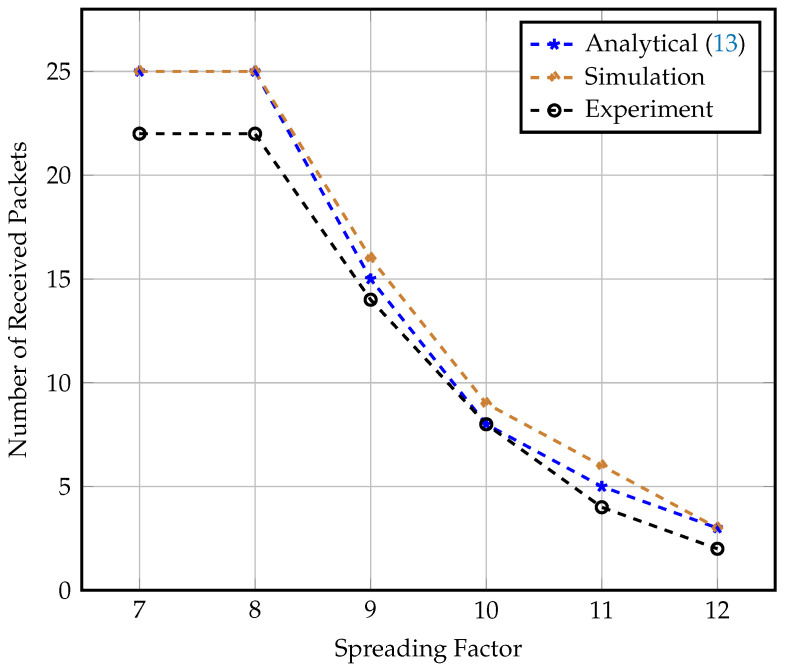
Number of received packets at speed = 90 km/h for different SF values.

Real experiment data, simulation data in ns-3 simulator, and analytical results are compared in Figure 8. The figure depicts the maximum number of packets received in real-world experiments and simulations, as well as the data from analytical expressions at various spreading factor values and a gateway speed of 90 km/h. The results show that the data from the experiment and simulation matched the expected analytical performance. The analytical data act as a lower bound for the remaining two curves. We notice that the level of received packets is constant for SFs 7 and 8 on 25 packets. This is due to the fact that the upper bound for possible received packets using these spreading factors is much higher than the 25 packets transmitted in this test. Furthermore, we find that for higher spreading factors, the number of received packets is reduced due to the limited visibility time and longer TOA values. We notice that for higher spreading factors, the data recorded in the real experiment are less than the analytical calculations, as expected, because the symbol time exceeds the channel coherence time.

### 4.2. Simulation of Gateway Arrival Scenarios

We continue the analysis of the three gateway arrival scenarios by running MATLAB simulation to investigate the effect of higher speeds on the visibility time, the resulting throughput, and energy consumption at the end-node. The throughput is presented as the number of received packets. For fairness of comparison, we test the performance for all spreading factors with a payload length of 25 bytes. The setup for all experiments and simulations is that the gateway height is 2.5 m, whereas the end-nodes are lying on the ground level, as depicted in Figure 3.

#### 4.2.1. The Synchronized Scenario

**Figure 9:**
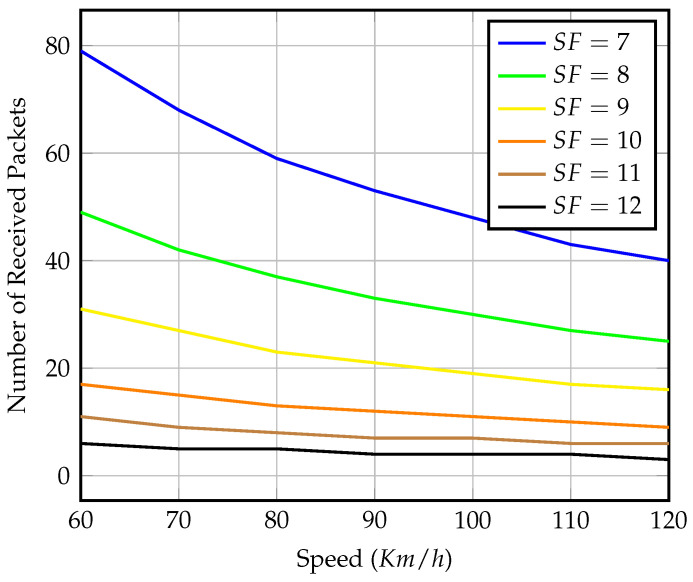
Number of received packets within effective visibility time of different speeds and SF values in the case of perfect gateway arrival synchronization.

Figure 9 depicts the number of received packets within the visibility time for each spreading factor at various speeds. The graph shows that in the scenario of precise synchronization, gateway mobility has no effect on LoRaWAN’s expected performance. The only advantage of using higher SFs is increased coverage. Even though a longer coverage distance means more visibility time, the transmission duty cycle constraint wastes this extra time. The number of packets received at each speed demonstrated is considered the upper bound for received packets at the same speed in later scenarios. For the remainder of this section, we will conduct our analysis with a fixed speed of 80 km/h as an average vehicle speed.

**Figure 10:**
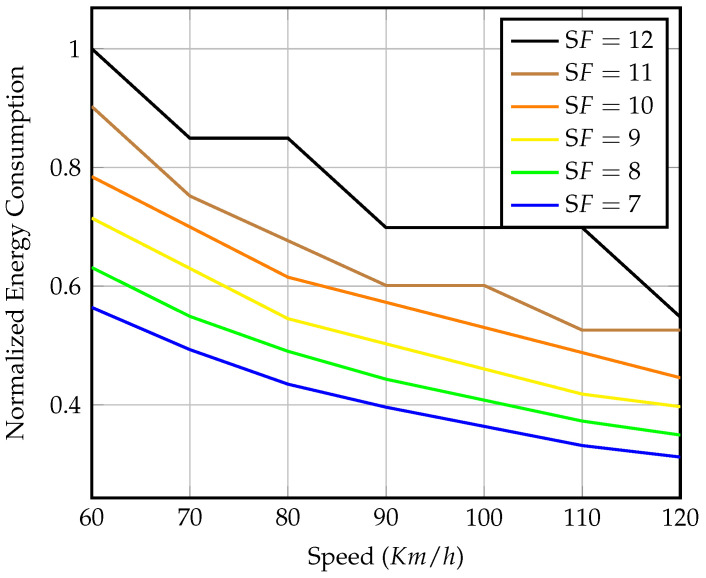
Normalized energy consumption of packets transmission for different speeds and SF values in the case of perfect gateway arrival synchronization.

Figure 10 shows the energy consumption relative to the throughput shown in Figure 9. As expected, the higher SFs consume more energy even though they transmit fewer packets due to their higher range and longer TOA, as explained earlier in Section 3.3. The selection of SFs transmission value must be optimized to meet a certain coverage distance, throughput limits, and minimum power consumption level requirements, especially for semisynchronized and nonsynchronized transmission, as discussed below.

Energy consumption values are normalized to a reference value, which is the energy of transmitting six packets using SF=12 with precise synchronization. The energy of transmitting using SF=12 with a gateway speed of 80 km/h is $E_{ref}=1.674J$ for each transmission window. This normalization will also be used for later scenarios for ease of comparison. For better presentation, for the rest of this section, we concentrate on the shortest SF=7, the longest SF=12, and the two in the middle. The missing spreading factors behave similarly within their maximum received packet limits.

#### 4.2.2. The Semisynchronized Scenario

**Figure 11:**
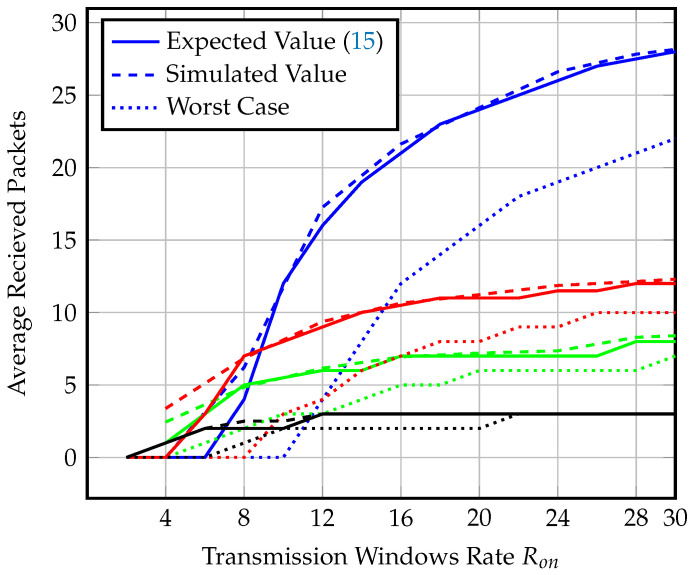
Average and worst-case number of received packets of different SF and $R_{on}$ values at speed = 80 km/h for semisynchronized transmission.

The end-node opens repeated ping slots, searching for the gateway at a rate $R_{on}$ per hour. We start with 4 windows per hour and increase up to 30 windows per hour. The number of windows are selected to guarantee gateway detection. The maximum synchronization delay that could be endured to detect the gateway presence is around 15 min using SF=12, and assuming a gateway speed of 60 km/h or less. We compare the analytical expression for the average number of received packets within effective visibility time (15) against the simulation results of 10,000 instances in Figure 11.

Figure 11 shows the average number of received packets for different spreading factors and different $R_{on}$ values at a gateway speed of 80 km/h. In order to investigate the limits on the system number of transmitted packets, we also investigate the worst-case scenario, where the gateway is only detected at the last ping slot, in this case, the end-nodes transmit the minimum number of packets. We find that the performance of the average throughput values for each SF comply with the synchronized transmission behavior; however, the worst-case scenario throughput of SF=7 drops for an opening rate of fewer than 15 windows per hour. We find that using SF=9 with $R_{on}=15$ windows per hour is optimal for coverage range, guaranteed throughput, and power consumption.

**Figure 12:**
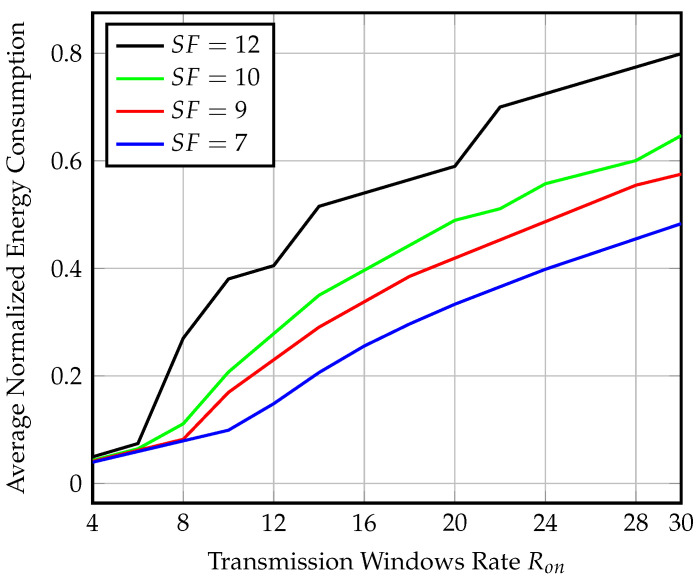
Average normalized energy consumption at the end-node for different SF and $R_{on}$ values at speed = 80 km/h for semisynchronized transmission.

Figure 12 shows the average normalized energy consumption for each $R_{on}$ for SFs=7,9,10, and 12 corresponding to the average throughput values in Figure 11. We find that the normalized energy consumption using SF=7 and, $R_{on}=30$ windows per hour is 0.48 for transmitting only 28 packets, which is fewer than half the number of transmitted packets with almost the same energy consumption in the synchronized scenario. The same performance is repeated for other spreading factors.

#### 4.2.3. The Nonsynchronized Scenario

Similar to the previous case, the end-nodes open ping slots at the same rate per hour $R_{on}$; however, the search for the gateway is repeated over several hours. We propose using multiple gateways for data collection to increase the gateway availability and enhance the freshness of data. First, we investigate the transmission with a single gateway.

**Figure 13:**
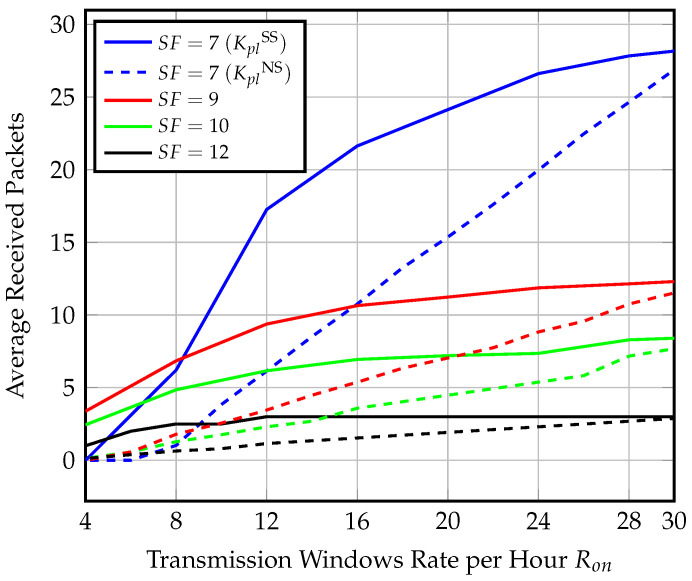
Average number of received packets of different SF and $R_{on}$ values at speed = 80 km/h and single gateway for nonsynchronized gateway arrival scenario.

**Figure 14:**
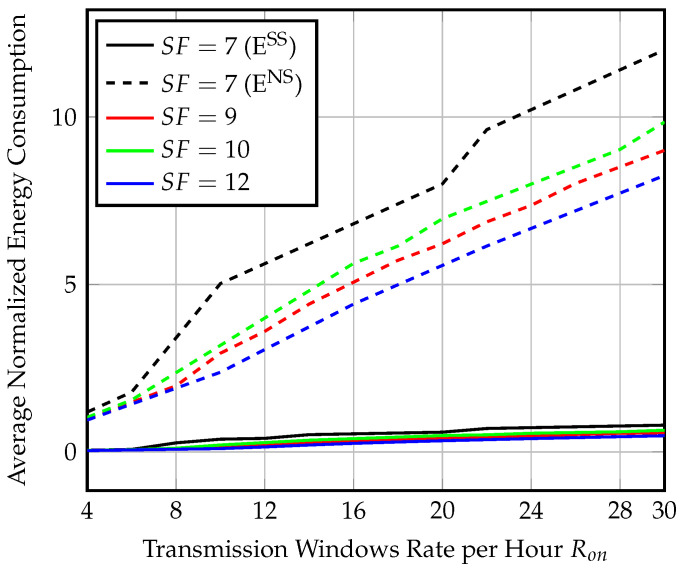
Average normalized energy consumption for different SF and $R_{on}$ values at speed = 80 km/h and single gateway for nonsynchronized gateway arrival scenario.

Figure 13 shows the average number of received packets for different SFs and, $R_{on}$ values. We compare the performance of semisynchronized transmission to that of nonsynchronized transmission with a single gateway. We notice the effect of no synchronization on the decrease in the number of received packets. To achieve the same values for average received packets of the previous scenario using a single gateway, we need to operate at $R_{on}=30$ windows per hour or more, at the cost of energy consumption at the end-nodes rising to up to 17 multiples for SF=7 and 15 multiples for SF=12, as shown in Figure 14.

**Figure 15:**
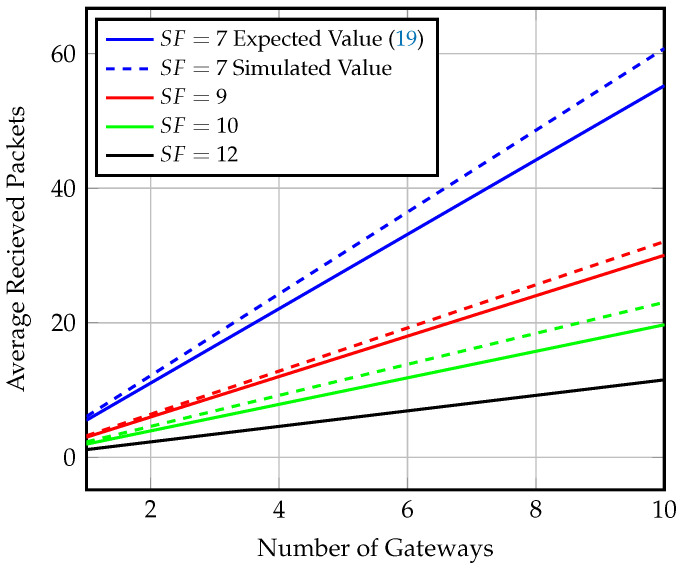
Average number of received packets for different SF values and multiple gateways at speed = 80 km/h and $R_{on}=8$ windows/h for nonsynchronized gateway arrival scenario.

**Figure 16:**
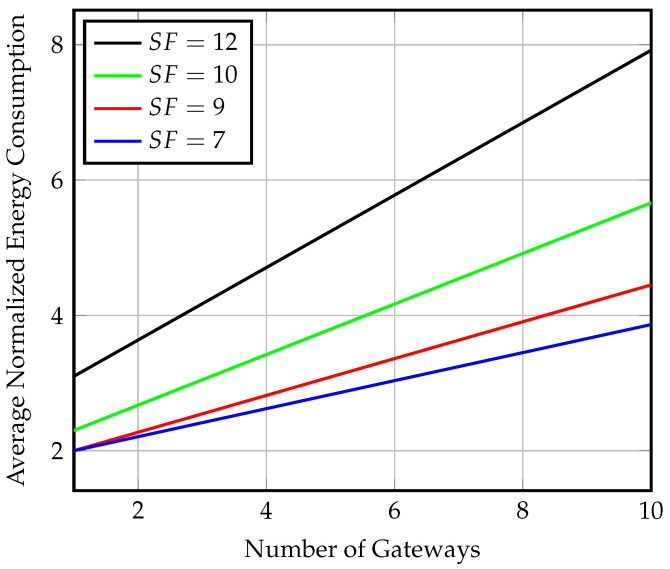
Average energy consumption at the end-node for different SF values and multiple gateways at speed = 80 km/h and $R_{on}=8$ windows/h for nonsynchronized gateway arrival scenario.

Using multiple gateways can significantly enhance the system’s performance in terms of throughput, energy consumption by end-nodes, and freshness of data, as the end-nodes will detect the gateway presence more frequently. Figure 15 presents the effect of increasing the number of gateways on the average number of received packets for different SFs at gateway speed of 80 km/h and $R_{on}=8$ windows per hour. The analytical values are compared to the results of a MATLAB simulation of multiple gateway arrivals. Figure 16 shows the effect of increasing the number of gateways on the energy consumption of the end-node. We observe an increase in energy consumption due to more transmitted packets as the probability of gateway detection increases.

Using four gateways increases the throughput achieved via SF=7 to four times the value achieved in the semisynchronized scenario for the same parameters of gateway speed and $R_{on}$ value. The number of received packets increases from 7 to 28 packets, with only five times the energy consumption when compared to using a single gateway. Data collection using multiple gateways with no transmission synchronization between the gateway and end-nodes can approach synchronized transmission performance. The synchronized transmission throughput of SF=7 is achieved by using ten gateways and only eight multiples of the synchronized transmission power consumption, while it would require 40 multiples of power consumption using a single gateway.

### 4.3. Wadi El-Gemal: A Case Study

In this section, we present a case study of Wadi El-Gemal national park in Egypt, a coastal area along the Red Sea in southeast Egypt (24.4747Â° N, 35.0987Â° E). We simulate the area with the ns-3^®^ simulator, distribute the end-nodes throughout the area, and preconfigure their transmission parameters in terms of SF allocation and transmission schedule to avoid collisions. We evaluate data collection performance using the synchronized transmission model.

**Figure 17:**
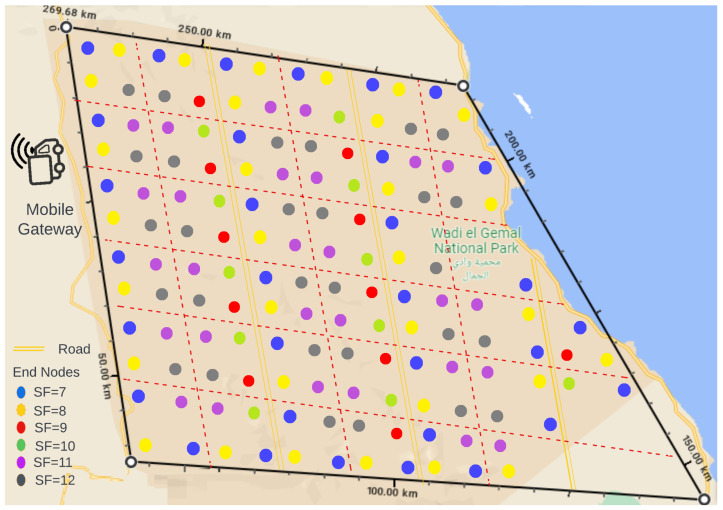
Distribution of end-nodes in the Wadi El-Gemal area.

The area shown in Figure 17 is 58×64 km^2^ area. Based on our analysis, we conclude that transmitting with SF=7 is optimal for end-node throughput and power consumption. Thus, we aim to use this spreading factor in most of the end-nodes. We intend to divide the area in such a way that there is no overlap in transmission time between successive nodes. The maximum communication distance using SF=7 is 7.4km, as stated earlier in Table 1. As a result, we decided to divide the area into 10×10 km^2^ subareas to ensure sufficient distance between the end-nodes operating with SF=7.

Each subarea is thought of as a cluster of four end-nodes. To cover the entire area, we use 146 end-nodes. The 146 distributed end-nodes and their associated spreading factor are represented by colored dots. The allocation of SFs takes into account two factors: first, the distance between the end-node and the gateway, and second, minimizing interference between successive end-nodes. The simulation parameters are the same values listed in Table 3.

The gateway collects data from one or two clusters as it passes by, depending on the location of the road. For example, the gateway collects data from four end-nodes along the circumferential road within the same visibility time. The two end-nodes closest to the road have spreading factors of seven and eight, respectively, while the two farthest end-nodes have spreading factors of 11 and 12. Whilst two clusters—one on each side—will also be transmitting at the same time along the vertical unpaved routes running across the middle area, spreading factors 7, 8, 9, and 10 were used alternately for the near end-nodes, while 11 and 12 were kept for the farthest end-nodes as the shorter ranges of lower spreading factors will not be sufficient to reach the gateway.

The simulation implements the synchronized transmission scenario, in which all end-nodes transmit continuously throughout the visibility time. The mobile gateway begins at position zero, with an initial distance of 2.5km from the first end-node. We assume having multiple gateways, with only one gateway passing by each node at speeds of 80 km/h. One gateway follows the circumferential road, while three others follow the middle routes, and each end-node transmits six packets of 25 bytes payload. Simulation results showed 100% packet delivery rate for all circumferential end-nodes and all end-nodes operating in SFs 10 and 11, while all end-nodes operating in SF11, as well as some of the end-nodes in the middle area, achieved PDR 83.33% due to one packet loss.

**Figure 18:**
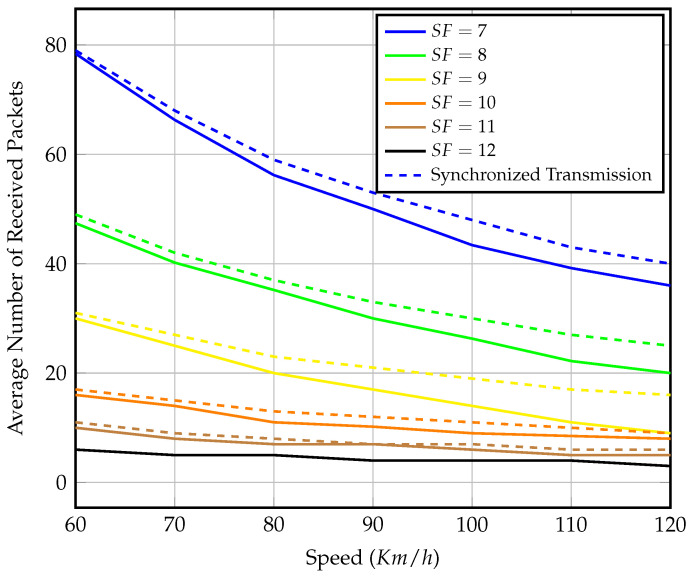
Average number of received packets per end-node within effective visibility time of different speeds and SF values.

To further test our setup, we repeat the simulation with different gateway speeds while setting the end-nodes to transmit continuously until the gateway is out of range. Figure 18 shows the average number of packets that the mobile gateway could receive from each end-node operating in a certain SF. We notice that the results are close to those of precise synchronized transmission shown in Figure 9, especially at lower speeds, and as the gateway speed increases, the visibility time decreases and the number of collisions increases.

## 5. Conclusions and Future Work

This work presents a monitoring system for rural areas and sensitive habitats that lack communication infrastructure. The system takes advantage of LoRaWAN communication’s long-range and low-power properties, as well as gateway mobility, to extend coverage with as little impact on the ecosystem as possible. The effects of gateway speed on the signal are discussed in terms of Doppler shift, range, and visibility time. The proposed system’s performance is analyzed, and three gateway arrival models are investigated. Finally, we provide a case study of monitoring one of Egypt’s most important national parks.

The three models of gateway arrival emphasize the trade-off between energy consumption and synchronization. Minimum energy consumption necessitates complete synchronization between end-nodes and gateway, which may necessitate complex transmission scheduling. While adopting a nonsynchronized transmission model would allow for more flexible transmission and a higher level of data freshness, it would also require more energy.

The results of our work show that the mobility of the LoRaWAN gateway limits the throughput corresponding to each SF due to limited gateway availability and transmission time. For synchronized transmission scenarios, using lower SFs provides better throughput; however, for semisynchronized and nonsynchronized transmission, higher SFs could ensure better performance as the longer visibility time allows for a higher probability of gateway detection. Data collection with synchronized transmission achieves almost 50% less power consumption compared to the semisynchronized transmission for the same number of received packets at gateway speed of 80 km/h.

Using multiple gateways with a nonsynchronized transmission setup enhances the freshness of data and simplifies transmission scheduling at the end-nodes. Nonsynchronized transmission with a single gateway consumes 15 times more energy than the semisynchronized scenario for SF=12 and 17 times more for SF=7. However, it can achieve the same throughput as that of a perfect synchronization scenario at the expense of eight times more energy consumption using 10 gateways for SF=7 and eight gateways for SF=12.

The proposed system is promising; however, further investigation of the optimum selection of LoRaWAN signal configuration is recommended. For future work, we will consider a more general case study on the implementation of our system, the optimal end-nodes placements, and the routing of the mobile gateway, as well as a data collection scheme for maximizing the sensing area and minimizing energy consumption.

## Figures and Tables

**Table 1:** Upper bounds of transmission distance and visibility time for each SF.

SF	SF7	SF8	SF9	SF10	SF11	SF12
$P_{\mathrm{rs}}$ **dBm**	−123	−126	−129	−132	−134.5	−137
**2$d_{\max}$ km**	7.4	8.4	9.4	10.5	11.5	12.6
**$T_{60\mathrm{km}/h}$ min**	7.41	8.40	9.42	10.50	11.52	12.61
**$T_{80\mathrm{km}/h}$ min**	5.55	6.30	7.05	7.88	8.63	9.45
**$T_{100\mathrm{km}/h}$ min**	4.44	5.04	5.64	6.30	6.90	7.56

**Table 2:** Detailed time duration and current consumption values for the power consumption model.

		Time Duration		Current Consumption	
	State	Variable	Value (ms)	Variable	Value (mA)
1	Wake-up	$T_{WU}$	168.2	$I_{WU}$	22.1
2	Radio preparation	$T_{RP}$	83.8	$I_{RP}$	13.3
3	Ping slot	$T_{PS}$	160.0	$I_{PS}$	$10\times 10^{-3}$
4	Transmission	$T_{X}$	$K_{pl}\times TOA$	$I_{X}$	83.0
5	1st wait window	$T_{W1}$	983.3	$I_{W1}$	27.0
6	1st $R_{x}$ window	$T_{RW}$	SF Dependant	$I_{RW}$	38.1
7	Radio off	$T_{RO}$	147.4	$I_{RO}$	13.2
8	Post-processing	$T_{PP}$	268.0	$I_{PP}$	21.0
9	Turn-off sequence	$T_{TS}$	38.6	$I_{TS}$	13.3
10	Sleep	$T_{S}$	$T_{ON}-T_{Active}$	$I_{S}$	$45\times 10^{-3}$

**Table 3:** Experiment and simulation parameters.

LoRaWAN Transmission Configuration				
$P_{t}=14$ dBm		$f_{c}=$ 868.3 MHz	BW = 125 KHz	SF∈ [7:12]
**LoRaWAN Packet Configuration**				
Payload	Preamble	Coding Rate	Low Data Rate Optimization	Header Mode
25 Byte	8 Byte	4/5	DE = 0 (disabled)	H = 0 (explicit)
**Path loss Model Parameters**				
Transmitted Power		Gateway Height	End-Node Height	Distance
$P_{t}=14$ dBm		$h_{m}$ = 2.5 m	$h_{b}=Ground$	$d_{0}=2.5$ km:$d_{max}$
